# Supplementary material for: How children and adults keep track of real information when thinking counterfactually
Source: PLoS One. 2020 Dec 4;15(12):e0242967. doi: 10.1371/journal.pone.0242967 (PMC7717521; doi:10.1371/journal.pone.0242967)
Supplement: S1 Appendix — In bold the correct response. (DOCX) [file pone.0242967.s001.docx]

**S1 Appendix. Example of the stories and percentage of correct responses (standard deviations in brackes). In bold the correct response.**

Imagine that you are a great detective whose mission is to investigate different cases. To do so, you have the support of your colleague Raúl who saw what happened and who will give you some information about it.

Hi, detective!

Today we have a lot of work. In each of the stories we have listened to part of the conversation between the police officer Raúl and other people will be our information. Pay attention to the words said by the policeman because he won’t repeat them. Will you be able to figure out what happened in each situation? Here are the cases, remember to pay attention to the information:

1. The police officer was at a birthday party and he saw a boy leaving the hall running. The police officer says that the boy is afraid of the sound of balloons bursting. You have to pay great attention to the police officer’s words because he gives you some information: remember that he says “the boy is afraid of the sound of balloons bursting”. Keeping in mind this information, could we say that there were balloons in the hall? Yes / No

Story 1- The police officer saw through a window that a child was in his bedroom and had a blank sheet of paper because his pen did not have ink. When he went out of his bedroom his sister went in and took the sheet and the pen. Later, the police officer says: “If/Even if his sister had used the pen…”

Counterfactual question- Would the sheet of paper have writing on it or would it be **blank**?’ Adults: 94 (*SD*= 23); Children: 78 (*SD*= 42)

Epistemic status question- Remember, the police officer said: “If/Even if his sister had used the pen …” According to this evidence, did the police officer see his sister using the pen? Yes / **No** Adults: 70 (*SD*= 46); Children: 49 (*SD*= 50)

Story 2- The police officer saw that a girl was on the beach playing with a bucket. Her bucket was broken and had a hole in the bottom. Later, the police officer said: “If/Even if the girl had poured water into her bucket…”

Counterfactual question- Would the bucket have been…full or **empty**? Adults: 89 (*SD*= 32); Children: 84 (*SD*= 36)
Epistemic status question- Remember, the police officer said: “If/Even if the girl had poured water into her bucket…” According to this evidence, did the police officer see the girl pouring water into the bucket? Yes / **No**  Adults: 63 (*SD*= 49); Children: 24 (*SD*= 43)

Story 3- The police officer saw that a girl was walking while eating an ice-cream. Then, the ice-cream fell on her shirt. She was walking near a puddle when a car passed. Later, the police officer said: “If/Even if the car had passed carefully…”

Counterfactual question- Would the girl’s shirt have been…clean or **dirty**? Adults: 80 (*SD*= 41); Children: 70 (*SD*= 46)
Epistemic status question- Remember, the police officer said: “If/Even if the car had passed carefully…” According to this evidence, did the police officer see the car splash the girl? **Yes** / No Adults: 70 (*SD*= 46); Children: 48 (*SD*= 50)

Story 4- The police officer saw that the floor of a boy’s house was wet because his mother had just washed it. When she finished, the boy came home from school and stepped in a puddle that was close to the door. Later, the police officer said: “If/Even if the boy had gone in barefoot…”

Counterfactual question- Would the floor have been…**wet** or dry? Adults: 85 (*SD*= 36); Children: 73 (*SD*= 45)
Epistemic status question- Remember, the police officer said: “If/Even if the boy had gone in barefoot…” According to this evidence, did the police officer see the boy go in barefoot? Yes / **No** Adults: 85 (*SD*= 36); Children: 54 (*SD*= 50)

Story 5- The police officer saw through a window that a girl was at home and the TV was turned off because it was broken. Her mother came into the hall to watch her favorite program. Later, the police officer said: “If/Even if her mother had turned on the TV…”

Counterfactual question- Would it have been…on or **off**? Adults: 89 (*SD*= 32); Children: 74 (*SD*= 44)
Epistemic status question- Remember, the police officer said: “If/Even if her mother had turned on the TV…” According to this evidence, did the police officer see the mother turning on the TV? Yes / **No** Adults: 65 (*SD*= 48); Children: 50 (*SD*= 50)

Story 6- The police officer who was at a swimming pool saw that a boy had an airbed. The airbed was deflated because it had been pricked. Later, the police officer said: “If/Even if his father had used an air pump…”

Counterfactual question- Would the airbed has been…inflated or **deflated**? Adults: 89 (*SD*= 32); Children: 79 (*SD*= 41)
Epistemic status question- Remember, the police officer said: “If/Even if his father had used an air pump…” According to this evidence, did the police officer see the father using the air pump? Yes / **No** Adults: 59 (*SD*= 50); Children: 35 (*SD*= 48)

Story 7- The police officer saw through a window of the room that a child was awake because his alarm clock had just sounded. His sister went into his bedroom to take a toy. Later, the police officer said: “If/Even if his sister had entered silently…”

Counterfactual question- Would the child have been … **awake** or asleep? Adults: 93 (*SD*= 26); Children: 76 (*SD*= 43)

Epistemic status question- Remember, the police officer said: “If/Even if his sister had entered silently …” According to this evidence, did the police officer see that his sister went in silently? Yes / **No** Adults: 87 (*SD*= 34); Children: 87 (*SD*= 33)

Story 8- The police officer was in a garden and saw that a child’s father went out shopping. When he came back, the car door was open because he had forgotten to close it. After that, the father went out and the mother went to the car to fetch some bags. Later, the police officer said: “If/Even if the mother had forgotten the car key…”

Counterfactual question- Would the car have been…**open** or closed? Adults: 91 (*SD*= 29); Children: 80 (*SD*= 40)
Epistemic status question- Remember, the police officer said: “If/Even if the mother had forgotten the car key…” According to this evidence, did the police officer see the mother with the car key? **Yes** / No Adults: 80 (*SD*= 41); Children: 58 (*SD*= 50)
