# Supplementary material for: How children and adults keep track of real information when thinking counterfactually
Source: PLoS One. 2020 Dec 4;15(12):e0242967. doi: 10.1371/journal.pone.0242967 (PMC7717521; doi:10.1371/journal.pone.0242967)
Supplement: Attachment — Submitted filename: Review.docx [file pone.0242967.s004.docx]

Overall, the manuscript has improved in clarity and readability. I still have some comments and questions, which I would like to see addressed:

Line 19/20. The sentence does not seem grammatically correct

Line 27. What is the take home message?

Line 51. Should this say “in the literature”?

Line 83. It is difficult to gage what it means that children “may not have understood them” – my intuition is that they simply process counterfactual questions differently from adults, but not that they do not understand anything. Careful framing is important here.

Line 317. I struggle to understand the difference between “representations” columns and “predictions to questions” columns. Is the point that adults represent both, while children represent only one situation? If so, I wonder whether this could be visualised in a more accessible way in the Table or elsewhere

Line 347 ff. Could these bullet-points style be transformed into proper text – it would help readability in my opinion.

Line 350. I am not sure I understand how the causal link is/isn’t made explicit.

Line 367. It is not quite clear to me based, on the manuscript, why only the representation for the antecedent seems crucial in identifying what is real and what is conjectured (at least, this is how I read this sentence). Is it because only the antecedent was provided in the question?

Line 384. Does the sentence in [] relate to [12] or to the current study?

Line 394/397. The switch from past tense to present tense seems a bit odd here.

Line 442/449. I am a bit puzzled that a difference of 7% is significant but a difference of 16% isn’t, despite seemingly similar SDs. Also Table 2 shows that numbers ending .5 are sometimes rounded up (89) while in Table 3 such numbers are rounded down (72).

Line 448. How can it be justified that responses from “even if” and “if” are averaged, when they have just been shown to be significantly different? Wouldn’t it make sense to make separate predictions for each in comparison to the epistemic status?

Line 475. Why is it probable that – if somebody can discard the conjectured model in the epistemic question – they can access that model in the inferential question?

Line 691. Not every researcher would agree that indicative conditionals are counterfactual – it is something philosophers would agree with, but developmental findings suggest there is a huge difference in the processing of indicative and subjunctive conditionals.

Line 714. Rafetseder & Perner (2010) show that reality biases are very rare beyond 4 year of age in counterfactual tasks. I doubt that a reduction in reality bias is the reason why children performed better in the current study.

Line 728. Could the authors be more precise in what they mean with “difficulty processing complex material”?

Line 766. What proposal are the authors referring to here?

Line 809. How could testing WM and ToM help shed light on the current results? This needs to be made clearer.
