# Supplementary material for: How children and adults keep track of real information when thinking counterfactually
Source: PLoS One. 2020 Dec 4;15(12):e0242967. doi: 10.1371/journal.pone.0242967 (PMC7717521; doi:10.1371/journal.pone.0242967)
Supplement: Attachment — Submitted filename: Response to Reviewers.docx [file pone.0242967.s005.docx]

RESPONSE LETTER

Please read our responses in red below Reviewer 1’s proposals.

Overall, the manuscript has improved in clarity and readability. I still have some comments and questions, which I would like to see addressed:

Line 19/20. The sentence does not seem grammatically correct

The sentence has been changed.

Line 27. What is the take home message?

The abstract explains the three sources of difficulty with counterfactuals. The take home message is that it is the epistemic factor and not the inference or the linguistic factor that explains children’s difficulty with counterfactuals. We made this explicit by adding “the third factor is critical: ….”

Line 51. Should this say “in the literature”?

Right. Done.

Line 83. It is difficult to gage what it means that children “may not have understood them” – my intuition is that they simply process counterfactual questions differently from adults, but not that they do not understand anything. Careful framing is important here.

We agree. We have changed the expression “they may not have understood them,” adding “in the same way as adults”.

Line 317. I struggle to understand the difference between “representations” columns and “predictions to questions” columns. Is the point that adults represent both, while children represent only one situation? If so, I wonder whether this could be visualised in a more accessible way in the Table or elsewhere

That’s right. Adults represent two models, and one question is about the “real” model while the second question is about the “conjectured” model. That is why in the prediction column, each prediction is aligned with the corresponding model in the representation column. We think that Reviewer 1’s difficulty in understanding was due to the spacing lines used. In Table 1, in the Basic interpretation row (children’s representation) there was no line spacing between “if” and “even if”, giving the impression that the representation for a conditional was two models (as Reviewer 1 detected). Now, with the changes in Table 1 in the basic interpretation row, “if” and “even if” have the same line spacing as in the “Mature” rows, and therefore, the one-model representation is visualised in a more accessible way.

Line 347 ff. Could these bullet-points style be transformed into proper text – it would help readability in my opinion.

Done.

Line 350. I am not sure I understand how the causal link is/isn’t made explicit.

There is a simple difference between Rafetseder et al.’s studies and our study. For example, they explicitly mentioned that a girl went into a room with mud on her shoes (cause), and the floor was dirty (consequence). In our study we do not explicitly inform about the consequence (participants need to infer it). Later, we question what would have happened if the cause had been present (would the consequence have happened?). We have included the example in the text.

Line 367. It is not quite clear to me based, on the manuscript, why only the representation for the antecedent seems crucial in identifying what is real and what is conjectured (at least, this is how I read this sentence). Is it because only the antecedent was provided in the question?

Yes, it is because the epistemic question in the task asks about the antecedent. The paragraph refers to the task’s differential predictions about how “if then” and “even if” are represented. The prediction is that the real and the conjectured models are the same (unlike what happens with the consequent).

In that paragraph, we added the text “referring to the consequent” and “tested by asking about the antecedent” to make this clearer.

Line 384. Does the sentence in [] relate to [12] or to the current study?

It is related to [12]. At the beginning of the text, we have added “In that study” to avoid ambiguity.

Line 394/397. The switch from past tense to present tense seems a bit odd here.

Agreed. Changed.

Line 442/449. I am a bit puzzled that a difference of 7% is significant but a difference of 16% isn’t, despite seemingly similar SDs. Also Table 2 shows that numbers ending .5 are sometimes rounded up (89) while in Table 3 such numbers are rounded down (72).

This is a misunderstanding: the percentages shown here were for the epistemic measure (average 72.5%) and the inferential measure (previously analysed in the previous paragraph; average 88.5%) just to show the great difference between these measures (16%). The analysis referred to in that paragraph as without significant differences is only in the epistemic measure as it is labelled in the epigraph (conditional variable: Even if vs If; that is 70% vs 75%). The text has been clarified in this respect.

Regarding the rounding, it may seem strange, but it is correct. It can be seen in the raw data; the two values are: 0.8866 (in text 88.5% and in table 89%) and .7245 (in text 72.5% and in table 72%). To avoid the same misperception by readers, we present data in the text without decimals as in the table.

Line 448. How can it be justified that responses from “even if” and “if” are averaged, when they have just been shown to be significantly different? Wouldn’t it make sense to make separate predictions for each in comparison to the epistemic status?

Please see previous point. There are no differences between “even if” and “if”, as the epistemic analysis showed.

Line 475. Why is it probable that – if somebody can discard the conjectured model in the epistemic question – they can access that model in the inferential question?

As mentioned in that section, two basic predictions of the mental model theory are that 1) adults represent counterfactuals with two models, and that 2) the labels of these models (conjectured and real) are easily lost, making it difficult to distinguish between the two models. If a participant correctly detects the “conjectured” model in the first (inferential) question (discarding the wrong epistemic one), it is because the label is accessible. In this case, the prediction of the theory is straightforward: that participants will access the epistemic model in the second question (because the label is accessible). This is more probable than the participant having taken the wrong model in the first question (because the label would not have been accessible).

*** From this point, the line numbering of the document referred to by Reviewer 1 corresponds with the document “with track changes”.

Line 691. Not every researcher would agree that indicative conditionals are counterfactual – it is something philosophers would agree with, but developmental findings suggest there is a huge difference in the processing of indicative and subjunctive conditionals.

We agree. There are well documented differences in the processing of indicative and subjunctive conditionals in children and also in adults. But we do not discuss here whether the use of the indicative to create counterfactual situations is appropriate. It is a fact. We just mention that some authors use factual conditionals to create counterfactual situations. The best example is Henry Markovits. We cite two of his studies on that point. To make the reading less “odd”, we have changed “counterfactual” to “contrary-to-fact” at this point.

Line 714. Rafetseder & Perner (2010) show that reality biases are very rare beyond 4 year of age in counterfactual tasks. I doubt that a reduction in reality bias is the reason why children performed better in the current study.

The term “reality bias” could be misleading, as Reviewer 1 mentioned, and has been changed. This term has been used (and questioned by some authors) to explain the difficulty of preschoolers in inhibiting knowledge about the reality (the actual outcome) in false belief tasks. Here we refer to the effect of presenting a result explicitly vs requiring participants to infer it. Only the fact of presenting that information has an effect on the probability of recovering that information, influencing their later responses (or judgments). As you know, this has been extensively studied and shown, for example, with the label of hindsight bias, in children and adults.

Line 728. Could the authors be more precise in what they mean with “difficulty processing complex material”?

In contrast with tasks used in other previous studies (22-24) with children under 5 that include few objects, this task includes stories with characters and actions that participants need to follow. The sentence has been changed: “the difficulty shown by schoolchildren is not due to an inability to think counterfactually per se, but it could be related to the difficulty in following counterfactual alternatives during the comprehension of stories with characters and actions”

Line 766. What proposal are the authors referring to here?

As we said at the end of the general discussion, “Neither Rafetseder et al.’s [12] results (p.401) nor ours can clearly discard the fact that children represent only one model; in fact in the present article we have left this possibility open to readers”. In the line referred to by the Reviewer, we mentioned a possibility referred to by Rafetseder et al. that the BCR is not the build model. This is consistent with representing just one model. However, that is not explicitly stated by the authors, and after reading a previous concern of Reviewer 1 (previous round), we have erased the sentence. Our argument does not need to attribute the proposal to Rafetseder et al.

Line 809. How could testing WM and ToM help shed light on the current results? This needs to be made clearer.

We have added the following lines to clarify the interest of the two measures: As shown, in other studies, preschoolers could solve counterfactual tasks with few objects and simple actions. In the present study, schoolers had some difficulties with counterfactuals tested with other tasks. They were given stories about people and actions and needed to keep track of the epistemic status of the possibilities. The working memory load and the need to attribute mental representation states in these tasks could have made the counterfactual inferences more difficult.
