# Supplementary material for: How children and adults keep track of real information when thinking counterfactually
Source: PLoS One. 2020 Dec 4;15(12):e0242967. doi: 10.1371/journal.pone.0242967 (PMC7717521; doi:10.1371/journal.pone.0242967)
Supplement: Attachment — Submitted filename: Response to Reviewers.docx [file pone.0242967.s006.docx]

All the corrections below proposed by the Editor have been made:

- Line 84: same way **as** adults ...
- Line 238: are expected **in** detecting ...
- Line 482: **was** to evaluate ...
- Line 488: this last aspect **had** not been ...
- Line 506: Additionally, we **aimed** to dissociate ...
- Lines 575-577: We carried out an ANOVA with Conditional (within-subject variable) and Age (between-subject variable) as the independent variables, and Inferential accuracy as the dependent variable.
- Line 648: **In** our research, ...
